# Ab initio chemical safety assessment: A workflow based on exposure considerations and non-animal methods

**DOI:** 10.1016/j.comtox.2017.10.001

**Published:** 2017-11

**Authors:** Elisabet Berggren, Andrew White, Gladys Ouedraogo, Alicia Paini, Andrea-Nicole Richarz, Frederic Y. Bois, Thomas Exner, Sofia Leite, Leo A. van Grunsven, Andrew Worth, Catherine Mahony

**Affiliations:** a Chemical Safety and Alternative Methods Unit, & EURL ECVAM, Directorate F – Health, Consumers and Reference Materials, Joint Research Centre, European Commission, Ispra, Italy; b Unilever PLC, Bedford, United Kingdom; c L’Oreal Research & Innovation, Aulnay sous bois, France; d INERIS, Verneuil-en-Halatte, France; e Douglas Connect, Basel, Switzerland; f Liver Cell Biology Laboratory, Vrije Universiteit Brussel, Brussels, Belgium; g Procter & Gamble, Egham, United Kingdom

**Keywords:** Ab initio, Safety assessment, Alternative method, SEURAT-1, *In vitro*, *In silico*

## Abstract

We describe and illustrate a workflow for chemical safety assessment that completely avoids animal testing. The workflow, which was developed within the SEURAT-1 initiative, is designed to be applicable to cosmetic ingredients as well as to other types of chemicals, e.g. active ingredients in plant protection products, biocides or pharmaceuticals. The aim of this work was to develop a workflow to assess chemical safety without relying on any animal testing, but instead constructing a hypothesis based on existing data, *in silico* modelling, biokinetic considerations and then by targeted non-animal testing. For illustrative purposes, we consider a hypothetical new ingredient x as a new component in a body lotion formulation. The workflow is divided into tiers in which points of departure are established through *in vitro* testing and *in silico* prediction, as the basis for estimating a safe external dose in a repeated use scenario. The workflow includes a series of possible exit (decision) points, with increasing levels of confidence, based on the sequential application of the Threshold of Toxicological (TTC) approach, read-across, followed by an “ab initio” assessment, in which chemical safety is determined entirely by new *in vitro* testing and *in vitro* to *in vivo* extrapolation by means of mathematical modelling. We believe that this workflow could be applied as a tool to inform targeted and toxicologically relevant *in vitro* testing, where necessary, and to gain confidence in safety decision making without the need for animal testing.

## Highlights

• A workflow for an exposure driven chemical safety assessment to avoid animal testing.
• Hypothesis based on existing data, *in silico* modelling and biokinetic considerations.
• A tool to inform targeted and toxicologically relevant *in vitro* testing.

## Introduction

Within the European Union (EU) innovations in the safety assessment of chemicals are required to support the EU policy to protect laboratory animals [30] and to provide new regulatory acceptable assessment approaches, especially after the full implementation of the EU Cosmetics Regulation [18]. Therefore the European Commission, within the frame of the FP7 Health Programme (https://ec.europa.eu/research/fp7/), together with Cosmetics Europe (https://www.cosmeticseurope.eu/) co-financed the research initiative “Safety Evaluation Ultimately Replacing Animal Testing (SEURAT)” (http://www.seurat-1.eu) in a public–private partnership [35]. The initiative was strongly inspired by the U.S. National Research Council report entitled *Toxicity Testing in the 21st century: A Vision and a Strategy* [68]. SEURAT-1 was planned to be a first step to address the long term strategic target, focusing on the replacement of animal testing in chemical assessment for repeated dose systemic toxicity. Six research projects and a coordination action contributed to the initiative, and combined the research efforts of over 70 European universities, public research institutes, and companies. The SEURAT-1 strategy [94] adopts a toxicological mode-of-action MoA) framework to describe how any substance may adversely affect human health [3], [8], [56] and uses this knowledge to develop complementary theoretical, computational (*in silico*), and experimental (*in vitro*) models that enable prediction of quantitative points of departure, needed for safety assessments [84]. The research initiative aimed to prove this concept on three levels [94], [95]: (1) theoretical descriptions of adverse outcome pathways (AOP) based on existing knowledge, (2) toxicity prediction based on hypothesis-driven testing employing *in vitro* and *in silico* methods, and (3) safety assessment applying existing information strengthened with selected data generated from alternative methods suitable for regulatory use.

SEURAT-1 undertook the *“ab initio”* case study by applying SEURAT-1 methods and approaches, as well as results from already existing alternative testing, e.g. ToxCast [29]. The aim was to develop a structured risk assessment workflow for repeated dose toxicity, with the goal of predicting a no-adverse effect level of a cosmetic relevant ingredient, assuming a certain exposure scenario. Within the context of this workflow, the Threshold of Toxicological Concern (TTC) approach, evaluated by the COSMOS project (http://www.cosmostox.eu/; [98], [99]) and refined for dermal exposure [97], was applied to support a low exposure scenario. In addition, read-across was incorporated to strengthen the non-animal evidence with structurally similar substances and make biological links to higher order outcomes [3], [8]. The application of the TTC approach and read-across was followed by a so-called “ab initio assessment”, meaning that the safety evaluation was carried out on the basis of hypothesis-driven *in vitro* testing combined with *in vitro* to *in vivo* extrapolation by computational modelling. While it was not considered realistic to fully complete such a risk assessment for a chosen substance within SEURAT-1, the case study is the basis for an integrated assessment that relies only on alternative methods. It showcases the feasibility of carrying out such an assessment, but also illustrates uncertainties and knowledge gaps. These learnings will assist in shaping a more focused strategy to advance alternative safety assessment approaches.

Within SEURAT-1, a conceptual framework for safety assessment was developed [13] outlining a logical basis for the different steps in a chemical safety assessment without performing additional animal testing. The conceptual framework was intended to provide the basis for the feasible design of integrated assessment approaches which can be adapted for a particular case depending on the purpose of the prediction, and the degree of uncertainty that can be tolerated. The overall outcome of an assessment based on the framework is anticipated to be robust as it is based on multiple pieces of evidence. Nevertheless the type and degree of uncertainty in the predictions needs to be understood to ensure that the assessment is ‘fit for purpose’.

The framework takes into account whether the substance is likely to exhibit general toxicity or a specific biological MoA. A large number of substances are assumed to provoke general toxicity [88], i.e. they tend to be ‘unselective’ in interacting with biological targets and hence have the potential for generic biological perturbation. Other substances, for example often in the case of pharmaceuticals or pesticides, are ‘selective’ in interacting with biological targets and have a known biological mechanism. Information on toxicokinetics and toxicodynamics are important in either case.

The safety assessment workflow developed here is based on the general SEURAT-1 conceptual framework. As stated earlier, applying the framework to an *ab initio* assessment at this point in time was a stretch goal aiming to highlight gaps for future development and illustrate overall progress made in SEURAT-1. It assists in structuring the information and provides guidance regarding what additional alternative data are needed to establish and then test a hypothesis. The assessment is based on gathering existing data and using information from alternative methods, as described in the guidelines for safety evaluation of cosmetic substances, which were developed and are regularly updated by the Scientific Committee on Consumer Safety [78]. We are here going further by organising the information into a logic workflow and by starting with exposure considerations so that both hazard and risk are incorporated into the *ab initio* assessment. Moreover our intention is that the workflow is general enough to cover any type of chemical and exposure, and need not be limited to cosmetics. The chemical to be assessed in the *ab initio* workflow can be a substance synthesized or extracted from natural source for the very first time or an existing challenged ingredient. The workflow could also be applicable to an already manufactured substance with a new intended use resulting in higher exposures that extends beyond previous assessments. The workflow starts from the same considerations regardless of the type of safety assessment. The starting point is Tier 0 where the exposure scenario and chemical identity are defined. This initial tier includes exit points where the TTC approach or a read-across assessment based on chemical similarity could be applied. In cases where neither of these approaches is considered to be adequate, it is necessary to proceed with applying the workflow. In the following steps, high throughput or high content data from alternative methods are collected under Tier 1 to better understand possible MoA, while Tier 2 is targeted testing based on the hypothesis(es) set up under Tier 1.

To illustrate this workflow a case study with a hypothetical exposure scenario was created for the substance x: a new ingredient introduced in a body lotion formulation, which is applied twice per day on skin (overall body surface).

## A workflow for chemical safety assessment with non-animal methods

**Fig. 1:**
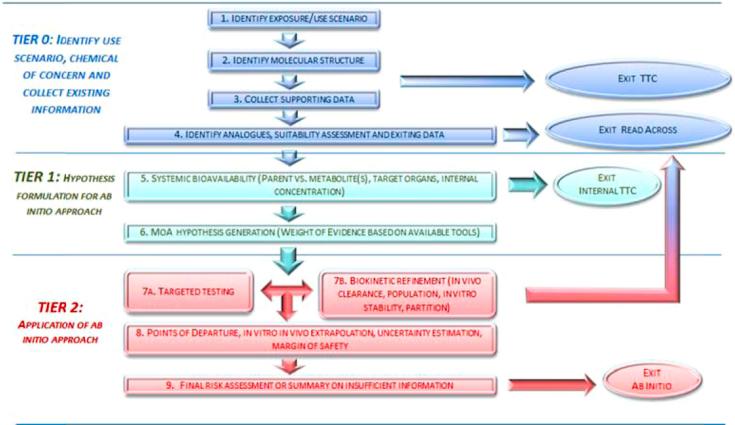
Workflow for the safety assessment of chemicals without animal testing.

We here outline a general workflow for chemical safety assessment (Fig. 1), based on the SEURAT-1 conceptual framework, but further elaborated, aiming to provide an tool to guide the assessor through the different steps to be considered and enable decision making. *Ab initio* means ‘from the beginning’; in the context of this workflow, *ab initio* assessment refers to the hypothesis-driven generation of new *in vitro* data and data interpretation (*in vitro* to *in vivo* extrapolation) by means of mathematical modelling. The more robust the information we manage to collect, the better it can assist us in making the hypothesis, and the better we are guided in identifying data gaps and elaborating a targeted testing strategy with a call for data as needed. We use existing human and animal data, when available, to underpin a hypothesis in combination with existing and generated *in silico* and *in vitro* data to provide the basis for targeted testing applying selected alternative methods. To provide confidence in the assessment, the level of uncertainty must be estimated for each step. If the uncertainty at the end is too large, the assessment will not be useful as such, but will be the basis for identifying the remaining gaps that are likely caused by lack of relevant and reliable methods. The workflow allows us to apply Thresholds of Toxicological Concern (TTC) or read-across approaches. These are indicated but not detailed further here as these approaches were described in other SEURAT-1 safety assessment case studies [6], [80], [97]. These approaches are treated as “exits” in the *ab initio* workflow, and it should be noted that they have already reached a certain degree of regulatory acceptance [19], [20], [21], [22], [76], [77].

The general workflow is illustrated in Fig. 1, and is step-wise described here below.

TIER 0: Identify use scenario, chemical of interest and collect existing information.

### Identify exposure/use scenario

If the chemical is part of a product and chemical release from the product matrix can be excluded, the chemical can be safely used because there is no exposure to the chemical from the product, e.g. exposure-based waiving under REACH [17] is applied. Of course it must be carefully evaluated whether there are any additional uses to be considered in the assessment when the chemical is or can become available during the lifecycle of the product (e.g. production and waste treatment of the product).

When describing the exposure scenario, it should be considered whether the exposure is intentional or not, and in both cases estimates of dose, expected routes of exposure, frequency and length of exposure, should be made. It might also be relevant to consider more than one source of exposure to the same chemical, even though this would not be requested in most current regulatory risk assessments. The concept of aggregate exposure is one that is gaining scrutiny [50], [53]. Another issue would be exposure to similar chemicals at the same time, due to for example a complex mixture, e.g. including similar hydrocarbons with different chain lengths. As an alternative to assessing them substance by substance, one could estimate their combined effect by applying concentration addition to the group of co-exposed chemicals assumed to have a similar effect.

In the risk assessment case study used here to exemplify the workflow, it was decided to investigate whether the use of a new ingredient x added into a body lotion formulation, can be safely used. The following hypothetical use scenario was applied: 12.5% content of x in a body lotion applied on whole body (female, 60 kg). An average exposure of a body lotion applied twice a day is estimated to 145 mg/kg/day (95th percentile of distribution for European consumers in Hall et al. [38]), which assuming 100% skin penetration corresponds to 18.1 mg/kg/day of x.

We would like to point out that chemical x is piperonyl butoxide (CAS No: 51-03-6) (PBO), that was selected because it fits into a chemical space relevant for cosmetics and is known to provoke hepatotoxicity in repeat dose oral toxicity studies [32]. We wanted to be able to apply methods developed within SEURAT-1, which in general had a strong emphasis on liver toxicity. In the case study we work with the assumption that PBO is a newly manufactured substance with unknown properties and that there are no animal data available for the substance. PBO is an example to illustrate and not to perform a comprehensive risk assessment.

### Identify chemical of interest

The molecular structure and structural variations (e.g. isomers) for the assessed chemical must be identified and if available with other chemical identifiers, such as CAS number, IUPAC name etc. It is important to identify and define the chemical structure fully and correctly. Free or commercial Software such as ChemSpider (www.chemspider.com) or ChemIDPlus are available as support. The use of chemical identifiers such as SMILES or InChiKeys are useful for identification and cheminformatics processing of the structure information.

Questions such as whether there are impurities or degradation or biotransformation products, in amounts considered of concern for toxicity, also need to be addressed.

It might be possible at this point to conclude by applying a TTC approach [10], [65], and not continue to a more refined assessment. The TTC is a risk assessment approach based on the concept that there is an exposure threshold below which there is a low probability of an appreciable risk to human health. If the chemical structure is not defined or genotoxicity cannot be excluded, an exposure below 0.15 µg/person/day would be considered in most cases a safe use. The TTC approach can be applied in cases of insufficient data, with some exceptions [23], [25], [79]. For a known structure, and when genotoxicity can be ruled out, the Cramer class can be derived and the estimated exposure compared to the TTC thresholds. Just like any risk assessment approach a reliable exposure estimate has to be available for TTC to be applied and the TTC values are usually based on oral intake, which is understood to be conservative as in most cases oral bioavailability is higher than via dermal exposure.

However, as in the case of cosmetics, the primary route of exposure might be dermal. Within the COSMOS project the TTC non-cancer dataset was enhanced with several hundred substances related to cosmetic ingredients [40], [99] and the applicability of the TTC approach was evaluated both for oral exposure [98], [99] and for dermal exposure, taking into account bioavailability differences between dermal and oral exposure [97].

Chemical x falls into Cramer Class III (1.5 µg/kg/day) and for the worst case scenario of 100% dermal absorption with 18.1 mg/kg/day, the TTC is exceeded by far.

### Collect supporting data

All available information on physicochemical properties for a substance need to be collected and can be of use at several points in the assessment procedure, if missing they would need to be measured. Physicochemical properties such as the molecular weight and partition coefficients (log P) may inform possible penetration through skin and other tissues. Several QSPRs (Quantitative Structure-Property-Relationships) for skin penetration have been developed on this basis (Dumont et al. [16], recent review of computational skin permeability models: Tsakovska et al. [90]). Volatility must be considered in the exposure scenario and determination made of whether it is possible to exclude intake through inhalation. If not, then it must be dealt with.

Based on accurate representations of chemical structure (including isomeric forms), one can apply one or more QSAR (Quantitative Structure-Activity Relationship) models. This will provide useful information on the potential biological activity profile of the molecule, and will give an indication of the potential for both selective a non-selective biological activity. *In silico* profilers can be used to identify active groups, such as functional groups that can bind to proteins or DNA. Selected physicochemical properties might also be requested as input to QSPR and QSAR modelling tools and where not available can be estimated or measured. Available software and databases can be used to obtain predicted (or existing measured) physicochemical properties, such as EPI suite [28], the CompTox dashboard (https://comptox.epa.gov/dashboard) or the OECD QSAR Toolbox (https://www.qsartoolbox.org) [70].

Furthermore, occurrence of toxicological active metabolites or detoxification by metabolism should be considered. There are a large range of simulators predicting possible metabolites [74]. One significant problem with these predictions is often that it might be difficult to understand which of the predicted metabolites would be actually formed and biologically active, and to which extent both in time and concentration they would be present at the target tissue. Based on prediction tools and evaluation of possible bioactivity of the metabolites, it could at this stage be considered whether certain metabolites need to be included or can be disregarded for the rest of the assessment. Based on knowledge of mode of action, it might be possible to evaluate the extent to which the identified metabolites are toxicologically relevant. It could also be recommended to perform testing of *in vitro* metabolic clearance at a later stage in the assessment.

Chemical compounds such as reaction products and metabolites, identified as of relevance to the assessment, need also to be subject to literature and data searching. Existing animal data available for any of the compounds should be collected and evaluated, as well as any data on human experience, e.g. through poisoning centers, clinical testing, biomonitoring or epidemiology studies. If the data are not available or not complete and robust enough for being applied in a risk assessment as such, it might still be supporting evidence in the hypothesis building and as well used in a final weight of evidence assessment together with alternative data.

In the case of chemical x, metabolism prediction with the OECD QSAR Toolbox v.3.0 pointed at the formation of metabolites with a potential to bind to proteins and DNA, as well as formation of possible reactive oxygen species (ROS). In addition to predict the potential metabolites, biological models with relevant metabolic competence must be chosen to achieve further data at higher tiers.

### Identify analogues, suitability assessment and existing data

In particular, existing data for compounds similar to the considered substance should be collected. To this aim, it is necessary to understand the chemical features of the molecule and similarity to other chemicals. For the structural chemical similarity assessment, it is important to identify which features are relevant and to decide on a suitable similarity metric (e.g. Tanimoto index, Rogers and Tanimoto [71], Johnson and Maggiora [47]). Information regarding chemicals with similar structure is an important first screening in order to identify substances with potentially similar MoA. However, chemical similarity alone is not sufficient, the biological similarity, including both toxicodynamic and toxicokinetic aspects and (bio)transformations has to be taken into account. A number of software tools are available to support finding suitable analogues, such as the OECD QSAR Toolbox, Analog Identification methodology (AIM), Toxmatch and AMBIT (see recent review in Patlewicz et al. [70]).

By grouping similar substances, it is possible to infer properties and toxicity from data-rich substances in the group (sources) to the data-poor ones (targets), or as in this case to a specific unknown target chemical, applying read-across. One of the key aspects of performing a read-across is the confirmation that the source and target substance belong to the same category and can be considered to be toxicologically similar. If in the weight of evidence later in the workflow additional proof substantiating the chemical and biological similarity and relevance of analogues is gathered, a read-across can still be considered and applied also in Tier 1 and 2 (see Fig. 1). A systematic strategy for formation and justification of a category of similar substances, as well as for assessing the uncertainty in the read-across argumentation, has been established in Schultz et al. [80] within the SEURAT-1 initiative.

**Fig. 2:**
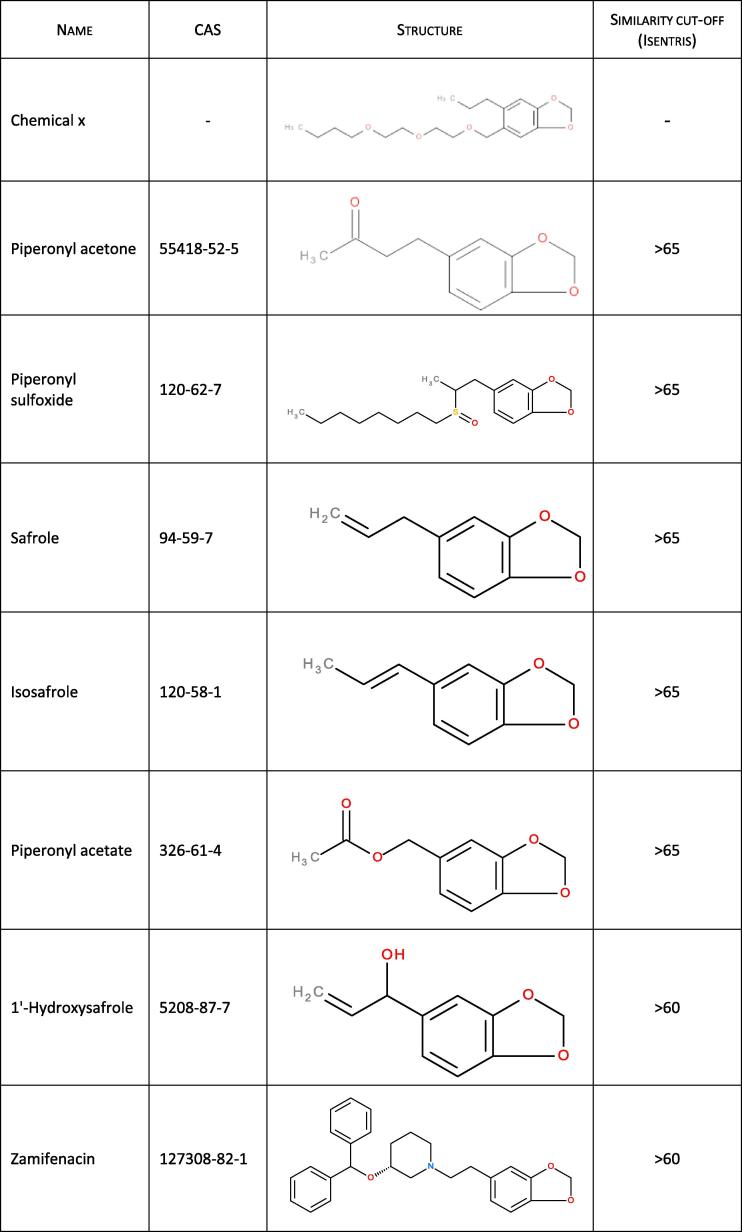
Alkenyl benzene compounds with similarity in molecular structure as compared to chemical X (kindly provided by Joan Fisher, The Procter & Gamble Company).

Fig. 2 includes molecular structures of alkenylbenzenes that have similarity to the core structure of chemical x. Isosafrole and safrole, 4-allyl-1,2-methylenedioxybenzene, are constituents that occur together in spices like nutmeg, mace and star anise and in some food products, such as root beer, pesto sauce, Bologna sauce [81], and are also used as natural flavouring. Both safrole and isosafrole are found to be rodent hepatocarcinogenes via both genotoxic and non-genotoxic mechanisms, respectively [43], [44], [45], [64]. None of the compounds illustrated in Fig. 2 are considered *similar enough* – or associated with sufficient data – to be used as a source substance for reading across existing data to chemical x, but they might still contribute in building the hypothesis for the *ab initio* approach [93]. One possible metabolic pathway is O-dealkylation at the aromatic system leading to ring opening and formation of catechol and guaiacol derivatives, could be similar to safrole and other compounds listed in Fig. 2, but compared to safrole and 1′hydroxysafrole, chemical x lacks the allyl group which is important for the metabolic activation needed to cause genotoxicity; additional differences in potential metabolic pathways would be based on the different side chain and read-across is therefore not applicable. If the read-across assessment is acceptable with sufficient confidence [22] at this stage, it would not be necessary to continue to Tier 1 of the *ab initio* workflow.

TIER 1: Hypothesis formulation for ab initio approach.

### Systemic bioavailability (target organs, internal concentration)

There are computational models that assist in predicting target organs that warrant further investigation. For example, predicting the probability that a certain compound can penetrate certain biological barriers, e.g. if a substance due to its properties cannot cross the blood–brain barrier, neurotoxicity could be excluded. Physiologically Based Kinetic (PBK^1^) models can be considered such models. PBK models are mathematical descriptions of interconnected compartments representing the human body, taking into account ADME (Absorption, Distribution, Metabolism, and Excretion) properties of a chemical within the body [12]. Application of such models will facilitate extrapolations across studies, species, routes and dose levels. These models are fundamental to the development of biologically based dose–response models to address uncertainty and variability related to kinetics and dynamics of a chemical. Once information is available, PBK models can be refined to facilitate the incorporation of the MoA by which a chemical is hypothesized to cause toxicity, resulting in a PBK-toxicodynamic (PBK-TD) model. As well as understanding if multiple compounds would be available at the same target organ at the same time and in what concentrations. Further considerations should then be made as to whether the chemicals could trigger the same molecular initiating event or a common key event leading to the same adverse effect [9].

**Fig. 3:**
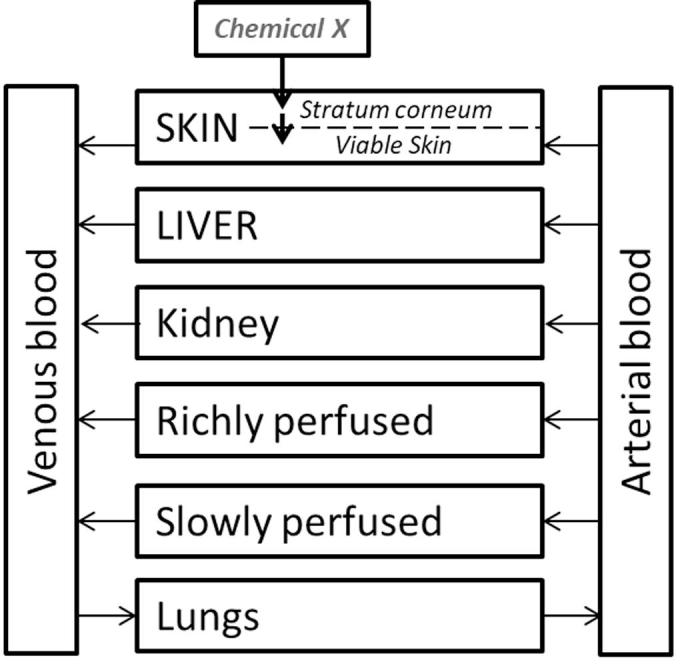
Schematic presentation of the physiologically based kinetic model used to simulate the distribution of chemical X within the human body.

The human PBK model, which was applied for chemical x (Fig. 3), was based on the human safrole model built by Martati et al. [61], [62], as safrole was identified (Fig. 2) to have a similar carbon skeleton as chemical x (but different functional group). A six compartment PBK model was developed, which included a skin compartment divided into viable skin and stratum corneum. Physiological and physicochemical properties were predicted using Dejongh et al. [14] as well as compared to safrole values [61] (Supplementary material 1, Table SM1). The exposure scenario for the body lotion containing 12.5% of chemical x, was applied to the PBK model. Monte Carlo simulations [7] were run to predict blood and liver concentrations in a population of 10,000 persons exposed daily to the body lotion. Parameter values were sampled randomly out of statistical distributions centred on the values given (Online resource 1, Table SM1). For the partition coefficients and the transfer rate from *stratum corneum* to viable skin we used lognormal distributions with a coefficient of variation (CV) of 300%. For hepatic clearance, the sole source of elimination considered, we had no information and used a uniform distribution ranging from 0 to the liver blood flow being the maximum possible hepatic clearance. The correlation between partition coefficient estimates was taken into account. From Monte Carlo simulation (10,000 iterations) output values were collected forming a random sample, for which we computed the mean, the standard deviation, and any percentile of interest. The predicted 95% confidence intervals for chemical x concentrations in the liver, blood and fat tissues of a consumer population is shown in Online resource 1, Figure SM1. The liver concentration range [1–100 µM] was used to establish doses to be tested *in vitro*.

There might be accessible data on realistic systemic doses obtained through biological monitoring or clinical data collected from, for example, human blood or urine, which may be useful to corroborate predictions but these may be difficult to relate to a particular exposure scenario.

### MoA hypothesis generation (Weight of evidence based on available methods)

Historical toxicity data on the substance of interest or structurally similar substances can provide a useful indication of the target organ(s) and help exclude certain modes of toxicity. In this case such data together with existing *in vitro* data and computational model results, would add to the weight of evidence to help focus on certain organs and identify the most relevant adverse outcome pathways (considering possible routes of exposure) that could be the basis for building a testing strategy. The number and variety of alternative test methods available is constantly growing. This technical progress is rapidly providing us with new biological models (for example, differentiated induced human pluripotent stem cells) that can provide cell systems for high throughput testing assays. ‘Omics techniques (e.g. transcriptomics, metabolomics, proteomics) collect large amounts of information, and the availability and interpretability of this data are rapidly growing. Alternative data sets are also becoming publicly available. Libraries are created with toxicity data for large sets of chemicals, as in ToxCast [29] or ‘omics databases, e.g. TG gates [46] and DrugMatrix (http://ntp.niehs.nih.gov/results/dbsearch/). These data, coupled with existing information from traditional animal-based studies can help to better understand linkages to adverse outcomes and can enable identification of new data that might assist in the safety assessment.

Screening of ‘omics databases, results from *in silico* profilers and *in vitro* libraries (like ToxCast) assist in providing a biological profile of the chemical. It could indicate whether there are general or more specific toxicity effects [13]. In case of a general toxicity profile, this information could be used to support a lack of biological effects, i.e. expected low toxicity. In addition Tier 1 data can include computational methods predicting various toxic effects from the data. Through QSAR models, the probability for certain hazard endpoints can be estimated, and data from *in silico* profilers can provide additional information on possible modes of action. These results can be considered together with the results from existing *in vitro* methods to strengthen the understanding of the mode of action. Consequently, based on prediction of Molecular Initiating Events (MIE) causing certain biological activity and provoking an adverse outcome in a related pathway, a better quantification of the dose necessary to cause an adverse outcome might be estimated.

Since we assume that no information of adverse effects is available for the specific compound x, a very large number of specific models or assays are needed covering a broad biological space to predict the MIE, as can be seen by the large efforts invested into the ToxCast [29] project. Therefore, an alternative way to identify areas of possible concern is needed, which then can target more specific assays. ‘Omics data provide a general overview of the molecular changes in a cellular system as a response to treatment with a chemical compound. Using bioinformatics and systems biology tools it should be possible to relate these changes to biological pathways and finally to possible adverse outcomes [36].

‘Omics data for chemical x were generated within the FP6-carcinoGENOMICS project and are available in the Data Infrastructure for Chemical Safety warehouse (diXa). The data represent three *in vitro* human hepatocytes models: HepaRG (human hepatoma-derived cells), HepG2 (hepatocellular carcinoma-derived cell line) and hES-DE-Hep (hepatocyte-like cells derived from embryonic stem cells). These models were treated with different concentrations of chemical x for 24 h or 72 h. For the pathway analysis, following partially the approach described previously by Kohonen et al. [52] on doxorubicin, we applied the Kyoto Encyclopedia of Genes and Genomes (KEGG) approach using the ‘Integrated analysis of Cross-platform MicroArray and Pathway data’ (InCroMap) software in order to select the most relevant molecular pathways influenced by the treatment with x. We observed important differences between enriched pathways in the HepaRG and HepG2 cells and non-specificity in the hESC DE-Hep cells. We also could observe dose- and time-dependent responses on all cellular models. The most enriched pathways (Q < 0.05) were then transferred and analyzed for genes, diseases and chemicals associations in the Comparative Toxicogenomics Database (CTD).

To identify adverse outcomes of chemical x, we initially followed a consensus approach by using the information of all three liver cellular models. The pathways enriched in at least two cell lines were selected and analyzed further using the CTD services in order to correlate the observed pathways with diseases showing that ‘dermatitis-allergic contact’ appeared as the most relevant adverse outcome. The skin sensitization would need to be further evaluated, but the indication of protein binding leading to skin sensitization also provide support for other repeat dose systemic toxicity, e.g. the formation of a reactive metabolite or activation of an immune response could also lead to hepatotoxicity. Since only seven enriched pathways were seen in at least two cell lines and a large amount of information could be lost, in a second approach we analyzed each dataset separately and looked at the sum of the identified effects. By performing this analysis, we showed that transcriptomics data were able to identify drug-induced liver toxicity as one major adverse outcome of treatment with chemical x. To cover a broader biological space additional cell lines/types should be tested to provide a truly comprehensive screen.

In order to support the identification or exclusion of target organs and the MoAs for human adversity and hazard characterization, a scan for possible effects can also be performed with *in silico* profilers, such as developed in the COSMOS project within the SEURAT-1 initiative. *In silico* profilers compile structural alerts or chemotypes (property-enhanced alerts) for specific endpoints/effects. Profilers for potential hepatotoxicity [39], protein binding and DNA binding [26], [27], mitochondrial toxicity [66], [67] as well as for phospholipidosis [75], also associated with liver toxicity, were applied. Potential Liver X receptor (LXR) binding was determined by employing and combining different *in silico* approaches, including ensemble docking, pharmacophore matching, fingerprint-based similarity and a QSAR classification model [31]. In addition, the potential for full PPARƴ agonism was predicted by a virtual screening procedure, including docking with filtering by four PPARγ pharmacophores [1], [89]. Other profilers for nuclear receptor binding were available to identify potential binding to the following nuclear receptors: PPAR, AR (androgen receptor), AHR (aryl hydrocarbon receptor), ER (estrogen receptor), GR (glucocorticoid receptor), PR (progesterone receptor), FXR (farnesoid X receptor), LXR, PXR (pregnane X receptor), THR (thyroid hormone receptor), VDR (vitamin D receptor) as well as RXR retinoic acid receptor) [65], [83]. Some of these receptors are associated with the development of hepatosteatosis. The results of the *in silico* predictions highlight nuclear receptor binding including PXR and PPARƴ. PXR is mainly expressed in liver, colon and small intestine, while PPARƴ is prevalent in adipose tissue. Other alerts were flagging carcinogenesis (http://www.proteinatlas.org/) and potential steatosis. Clearly the *in silico* profilers do not represent a complete breadth of possible repeat dose toxicity targets and so a weight of evidence approach is taken to filter down the scope to include the critical MIE and their associated AOPs or MoA that need to be further refined and quantitated through a dose response relationship to inform the risk assessment. In this case the use of orthogonal lines of evidence coming from the *in silico* alerts and *in vitro* data have been aggregated and viewed in relation to the outlined PBK model to define a logical and scientific rationale for those responses likely to be most relevant.

Chemical x was also analyzed across the multiple ToxCast assays as part of the Phase I testing [15]. This data have been made publically available through the Toxcast Dashboard https://actor.epa.gov/dashboard2/). 92 active assays, out of the 700 examined, were identified based on the structure of chemical x. These showed activity across a broad concentration range from 0.1 µM to 100 µM in cell free assay of different cell types. Further filtering has been applied to reduce this assay set to a subset of 26 assays believed to be most relevant to MoA determination. The selection was made on human relevance (removal of rat CYP NVS assays), removal of cytotoxicity assays and application of the cytotoxicity burst consideration as described in Judson et al. [49]. This defines a z-score derived from 3 median absolute deviations from the median cytotoxicity response. The authors noted that the *in vitro* assay responses could be delineated into two categories; specific and non-specific responses, with the former occurring at lower concentrations and separated from the non-specific responses that seem to be associated with cellular stress around the cytotoxicity limits of the compound. This provides a focus on those responses deemed to be specifically related to chemical x perturbation.

Furthermore this data highlights the sensitivity of the CYP metabolism proteins to chemical x with CYP2J2, 3A4, and 2C19 with the lowest AC_50_ = 0.1, 0.5 and 1.5 μM, respectively. For four additional P450 enzymes, perturbations are predicted to occur below 10 μM. It should be noted that these assays were performed in a cell free environment and therefore would more likely have a higher sensitivity, however further support of the impact of the compound on metabolic processes can be observed in the transcriptomics data. Here the sensitivity of a metabolically competent cell line, HepaRG, indicated by the pathway level transcriptional changes, is greater than for a cell line with low level of metabolic competency, HepG2, i.e. 3.2 μM vs. 45 μM at 24 h.

Simulation with the PBK model provides an indication that there is a higher exposure within adipose tissue and kidney rather than liver. However looking broadly at the weight of evidence that the more sensitive biological responses observed are present in metabolically active tissues such as liver, alongside strong indications of specific key events related to liver AOPs, these suggest that the liver is the primary target organ of concern. The predicted accumulation of chemical x in adipose tissues resulting in higher exposure combined with supporting weight of evidence from publically available data in the human protein atlas (http://www.proteinatlas.org/) highlight prevalence for the PPARƴ receptor within adipose and liver tissues. Therefore besides hepatotoxicity, effects in adipose tissue would need to be further addressed. Effects on kidney cannot be ruled out at this stage, given the predicted accumulation of chemical x there. A preliminary investigation on renal features like organic and cationic transporters as well as on renal epithelial cells is warranted to evaluate the effect of chemical x on kidney [11].

While these initial biomarkers provide some support for potential AOPs and associated tissues that can be identified, it is clear that the scope of the assays and the outlined AOPs (https://aopwiki.org/aops/34, https://aopwiki.org/aops/38) does not currently cover all biological functions and toxicological endpoints. The transcriptomics data provide some supporting evidence e.g. a relevant concentration to use for a point of departure, or biological pathway altering dose [48]. Further refinement to confirm a point of departure can be done within more complex test systems that enable an examination of changes due to multiple dosing and across longer time frames accounting for potential accumulation or secondary biological cascades.

TIER 2 Application of ab initio approach.

### Targeted testing and biokinetic refinement

The choice of methods to apply should be based on the MoA hypothesis. Some of the methods can be chosen because they are expected to confirm a certain effect, and others to demonstrate the absence of an effect. In addition it is useful to include some sort of cost-benefit analysis, and to set up a testing strategy that is able to provide evidence without applying all available methods.

To benchmark the applied experimental system it is necessary to include substances with known toxic effect (positive control) as well substances without might be helpful (negative control). This provides a basis of relevance and reliability in the experimental set up, to better define concentrations necessary to achieve a positive effect in the exposed cells and estimate potency. It is necessary to observe both positive and negative effects at different concentrations, to verify the competence of the system, and to repeat every experiment in order to confirm statistical reproducibility.

#### Targeted testing

When indications on target organs/tissues are obtained from the previous step, specific methods can be considered to derive a quantitative (dose–response) estimate of biological effects including doses corresponding to realistic human exposure. The alerts gathered from *in silico* and *in vitro* testing in tier 1 may point to known and well described AOPs. In such case, the focus is directed in investigating the key events of the selected AOP(s).

Different tools are available to address such targeted testing: from 2D based assays to organotypic models, including the microphysiological systems. Publications covering different types of assays are available [5], [33], [60], [77], [85]. The choice of relevant assays is based on the specific concerns that need to be addressed. In case “selective” biological effect(s) are identified, e.g. binding to a given nuclear receptor, the targeted tissue/organ, abundance and pathways where such “biomarker” is involved should be sought. It informs the type of biological model and level of complexity needed (single cell type, co-cultures, monolayers or 3D systems, static or dynamic systems) for the targeted testing. Spheroids and organ-on-a-chip models carry the promise of being more physiologically relevant than monolayer cultures, allowing repeated treatments over an extended period of time [96]. For “unselective” compounds, generic cell function [82] can be investigated with appropriate models [59], [63], [91]. As discussed below, the treatment frequency, duration and dosimetry at this step are to be carefully considered through refined kinetic modelling approaches.

In the case of chemical x, the *in silico* and broad screening testing (ToxCast and transcriptomics data) pointed to binding of PPARƴ, PXR, activation of a subset of cytochrome P450s and biotransformation pathways, leading to potential liver toxicity. PXR is mainly expressed in liver, colon and small intestine, while PPARƴ is prevalent in adipose tissue. Based on these alerts and the tissue localization of the PPARƴ and PXR, targeted testing is warranted for both liver and the adipose tissue. In addition, Zamefenacin, a chemical with similar structural elements (see Fig. 2), is known to cause hepatotoxicity in species able to metabolise it into an O-methylated derivative [2].

The targeted testing was based on assays developed in SEURAT-1 (http://toxbank.net/, https://ecvam-dbalm.jrc.ec.europa.eu/; [86]), Table 1. Both the steatosis and fibrosis AOPs were investigated ([41], [57], [58], [92], https://aopwiki.org/aops/34, https://aopwiki.org/aops/38). The adipose tissue and kidney were not evaluated, since they were not part of the SEURAT-1 toolbox, although the kinetics modelling suggested accumulation of chemical x in adipose tissue. Furthermore, from high throughput screening data available in ToxBank, there is an indication that there might be a concern for liver steatosis, due to lipid droplet accumulations.

**Table 1:** List of *in vitro* and *in silico* methods from the SEURAT 1 initiative considered in this case study.

	Method	Comments	Reference	Reported in DB-ALM [86]	Project
1	TTC approach	Oral to dermal extrapolation	Williams et al. [97]	N/A	COSMOS
2	Read-across approach		Schultz et al. [80], Berggren et al. [6], ECHA [20], [21], [22]	N/A	COSMOS
3	PBK model → chemical X concentrations		Martati et al. [61], [62], Bois et al. [7], see Online resource 1 https://knimewebportal.cosmostox.eu	Method Summary No. 161	COSMOS
4	‘Omics	*in vitro* human hepatocytes models: HepaRG (human hepatoma-derived cells), HepG2 (hepatocellular carcinoma-derived cell line) and hES-DE-Hep (hepatocyte-like cells derived from embryonic stem cells)	Omics data analysis as in Grafström et al. [36] based on data from FP6-carcinoGENOMICS project, diXa Warehouse (http://www.dixa-fp7.eu)	NO	ToxBank
5	High throughput /content screening	*in vitro* human relevant cell lines	EPA (US Environmental Protection Agency) [29] ToxCast™ Data, National Center for Computational Toxicology. http://www.epa.gov/ncct/toxcast/data.html	N/A	ToxCast™
6	PPARγ full agonism prediction	virtual screening procedure including docking with filtering by four PPARγ pharmacophores	Tsakovska et al. [89], Al Sharif et al. [1]	Method Summary No. 168	COSMOS
7	Prediction of potential LXR binding	Prediction combining different *in silico* approaches	Fioravanzo et al. [31] Model available through https://knimewebportal.cosmostox.eu	Method Summary No. 169	COSMOS
8	*In silico* profiler for Nuclear receptor binding	AHR, AR, ER, FXR, GR, LXR, PPAR, PR, PXR, RAR, RXR, THR, VDR	Steinmetz et al. [83], Mellor et al. [63] Model available through https://knimewebportal.cosmostox.eu	Method Summary No. 177	COSMOS
9	*In silico* profiler for potential hepatotoxicity		Hewitt et al. [39] Model available through https://knimewebportal.cosmostox.eu	Method Summary No. 179	COSMOS
10	*In silico* profiler for protein binding and DNA binding		Enoch et al. [26], [27] Models available through https://knimewebportal.cosmostox.eu	Method Summaries No. 181, 178	COSMOS
11	*In silico* profiler for mitochondrial toxicity		Nelms et al. [66], [67] Model available through https://knimewebportal.cosmostox.eu	Method Summary No. 180	COSMOS
12	*In silico* profiler for phospholipidosis		Przybylak et al. [75]	N/A	COSMOS
13	*In vitro* organoid co-culture of HepaRG with human stellate cells	*In vitro* → fibrosis	Leite et al. [60]	NO	HemiBio
14	VCBA		Zaldivar et al. [100], [101], [102], [103] Model available through https://knimewebportal.cosmostox.eu	Method Summary No. 162	COSMOS
15	IVIVE		Gajewska et al. [34] Model available through https://knimewebportal.cosmostox.eu	N/A	COSMOS
16	HTS/HCI	*In vitro* → Cytotoxicity using cellomics, single exposure (24 h)	Not published	Method Summary No. 167	COACH/JRC
17	HTS/HCI	*In vitro* → Cytotoxicity using cellomics, multiple exposure (4 * 6 h)	Not published	Method Summary No. 167	COACH/JRC

**Fig. 4:**
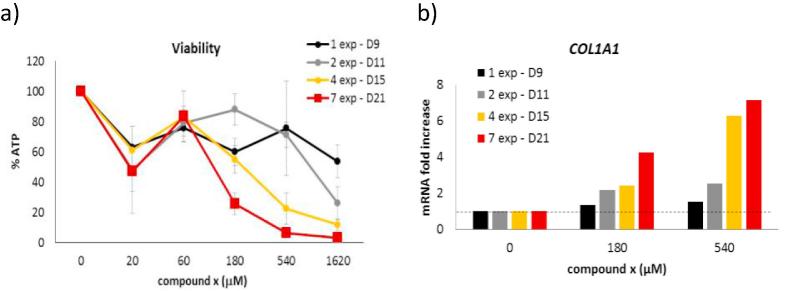
Fibrosis evidenced in hepatic organoids (3d HepaRG/HSC). a) Viability determination of organoids after 24 h exposure of chemical x after 1, 2, 4 and 7 exposures. b) D) mRNA levels of HSC activation marker COL1A1 in hepatic organoids after 1,2,4 and 7 exposures of 180 and 540 μM PBO.

To assess the fibrotic potential, compound x was tested in repeated dose, on human hepatic organoids composed of HepaRG and human hepatic stellate cells (HSC) [60]. Culture maintenance and exposure was performed as previously described in Leite et. al. Biomaterials [60]. After a week of cultivation, the cells were exposed every second day to a series of concentrations of compound x (20–1620 μM) during 2 weeks (D8 to D21). Cell samples were collected 24 h after exposure on days 9, 11, 15 and 21 to check cell viability (ATP) and gene expression of HSC activation markers. Results show that the compound is barely toxic after 1 exposure (Fig. 4a – D9), nevertheless dose–response toxicity increases over-time. To analyse gene expression, concentrations selected were the ones where an effect is observed on viability results, but not completely killing the cells, thus the 180 and 540 μM (Fig. 4b), using *COL1A1* as a reference marker for HSC activation. It can be seen that both concentrations activate HSCs and this activation increases upon repeated exposure. Only after the 4th exposure a dose-dependent effect can be observed, which might be related with the uptake of the available dissolved compound by the cells.

For time sake, these experiments were run independently of a dosimetry refinement by kinetic modelling, although chemical x was dosed in medium with or without cells. This provides a better estimate of an “effective” concentration rather than using the nominal concentration (some compounds may bind to the tissue culture material and/or the medium and may not be available for the cells to elicit a biological effect). Ideally, these assays should be run after such refinement and considering the lowest biological effects triggered at 1.6 μM in the transcriptomics study.

Furthermore, cells were exposed in parallel, Methotrexate, a known pro-fibrotic compound, which has shown a profile similar to compound x (data not shown).

#### Biokinetic refinement

In parallel to the testing strategies, it may be necessary to consider further refinement of exposure, both an estimation of the internal dose at the possible target organs as well in the test systems themselves. Through PBK models it is possible to calculate internal dose (internal concentration) from external exposure and predict a more realistic dose/concentration in the *in vitro* experiments. Biokinetic models can also be applied to translate and extrapolate *in vitro* experimental concentration to simulated intracellular concentration. Several reports have highlighted the concerns of extrapolating *in vitro* results based on the nominal concentration of a chemical applied to the *in vivo* situation where a free concentration of compound metric is applied [37], [55]. It is clear that such an extrapolation can lead to significant both over and underestimation of the physiologically relevant dose needed to cause an effect.

The Virtual Cell Based Assay VCBA), [103] is a mathematical tool developed within the COSMOS/SEURAT-1 project to simulate the fate of the chemical in an *in vitro* assay and was applied to obtain the dissolved concentration of chemical x that could enter the cell. The VCBA model has similarities to the Armitage model [4] and similar as the one developed by Kramer et al. [54]. The relevant parameters for running the VCBA can be estimated based on *in silico* predictions, using tools like EPI suite [28] or databases like Chemspider (http://www.chemspider.com/), or the CompTox dashboard (https://comptox.epa.gov/dashboard). The *in vitro* concentration response curve to optimize the VCBA model can be taken from experimental cytotoxicity testing. Information needed to perform the VCBA simulation for chemical x (Based on Zaldivar et al. [100], [101], [102], [103] is listed in Table SM2 Supplementary material. In the present case it indicates that the freely available fraction of the compound in the *in vitro* cell line assays not bound to serum, lipids or plastic is less than 5% of the nominal concentration (see Table in SM), this shows that not more than 5% of chemical x is available to be uptake by the cell.

It is further necessary to evaluate which is a relevant exposure scenario to apply to the testing – a single (one) exposure or repeated exposure. It might be that a chemical can accumulate in the cells of the target organ. Elimination rates must therefore be estimated. It is also important to understand if there is a maximum concentration (C_max_) causing toxicity, regardless whether it is occurring after one dose or repeated doses reaching the same dose after accumulation, or whether a repeated disturbance of the system with many non-toxic doses will lead to adverse outcome, even though no accumulation in the cell occurs (AUC; area under the concentration–time curve). In other words, an assumption needs to be made regarding the “structure” of the toxicodynamic model, which could be based on C_max_, AUC, or on a combination of these two parameters. In the translation of the *in vivo* system to the *in vitro* model, it must be noted that the time scale might be completely different due to the limitations in metabolic activity in the *in vitro* system. Therefore the repeated challenge mimicked in a short time interval might be more relevant.

Further refinements to the relative skin absorption may be possible using the tiered decision tree that was developed within the COSMOS project based on estimated skin exposure and dermal absorption [97]. Based on this decision tree for TTC, with a percutaneous absorption of 2.1% (calculated using J_max_, [42] and Systemic Exposure Dose formulas), confirmed by *in vivo* literature data in the COSMOS skin permeability database for chemical x, a maximal systemic availability of 380 µg/kg/day is calculated. Neither in this case of refined dermal exposure scenario can the TTC approach be applied to our x example. Using TTC, a concentration of 0.0493% in a body lotion could be supported. Furthermore, skin absorption in TIER 1 was assumed to be 100% however after refinement of the absorption this was applied to the PBK model and simulation for chemical x resulted in concentration two orders of magnitude lower when compared to the 100% absorption (SM Fig. SM1). In general, exposure data can inform *in vitro* exposure conditions, ensuring that dose and time are properly spanned with appropriate inflection points.

### Points of departure, *in vitro-in vivo* extrapolation, uncertainty estimation

The final steps in the chemical safety assessment workflow would be:

• Prediction of a point of departure for safety assessment based on the relevant AOP incorporating kinetics and biomarker data from repeated dose assays
• Definition of the margin of safety based on variability and uncertainty estimates
• Description of the safety decision and any open issues that could assist in gaining higher confidence.

**Fig. 5:**
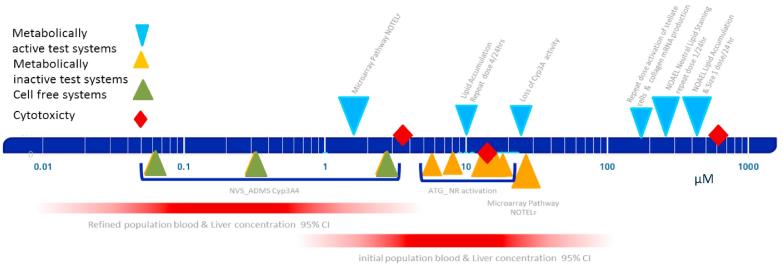
Illustration of predicted liver and blood concentrations of chemical X alongside *in vitro* assay results overlap. Differences in dose response are seen between the different test systems, e.g. Cyp3A4 an approx. 100 fold difference in concentration between cell free and the metabolically active HepaRG cells. This needs to be taken into account when translating the data for *in vivo* relevance. These data could be considered as possible points of departure for chemical x. Reprinted from OECD [69].)

The new data collected (Fig. 5) can confirm our hypothesis and elucidate points for departure for a quantitative risk assessment, or it might provide different evidence from what we expected. In the latter case it is necessary to carefully analyze the results to understand if the unexpected results, in magnitude or nature of the effect, were due to how the testing was performed. If the internal exposure is estimated to be far below any biologically active dose based on results from relevant *in vitro* assays, it could be concluded for that there is no concern for toxic adverse effect. However, depending on the confidence in the outcome and its relevance to a certain adverse effect, it might be considered that additional proof is needed to confirm the effect level.

*In vitro* testing results could be processed using the benchmark dosing approach to gain a point of departure for risk assessment that considers the overall concentration response curve, Benchmark Concentration (BMC). Additionally by applying *in vitro* data and *in silico* predictions we need to extrapolate back to the human situation, to reduce or avoid the uncertainty factors we can apply interoperable mathematical models like PBK and VCBA. The VCBA is one such a tool to perform this extrapolation to account for *in vitro* dosimetry effects and at the same time can be used to calculate the intracellular concentration in the *in vitro* test system to the actual *in vivo* dose. Thus, concentration of the chemical in the target organ (or in the blood) can be related to the intracellular concentration available in the cells in the test system or unbound chemical in the culture medium. In addition, internal exposure is translated into a realistic external exposure through an *in vitro* to *in vivo* extrapolation (IVIVE), and this should ensure that the worst case dose is accounted for in the testing. This is a refinement from considering the solubility limit of the chemical as the highest dose, which might be orders of magnitude different from the IVIVE extrapolation. Applying the VCBA simulations in combination with the PBK model can be used to perform IVIVE [34]. This will allow understanding of the external exposure needed to initiate an effect. The effect (for example cytotoxicity) is linked to the intracellular concentration simulated by the VCBA and used as input into the PBK model, which will scale up to the full body and translate the intracellular/organ concentration to an external dose. The PBK model can also be refined to simulate more endpoints, such as DNA adduct formation [73], [76] or cytotoxicity using impedance data [87].

Before progressing further, i.e. to trigger new testing or to the last step of the workflow, all data analyses should be completed. It is most important that the uncertainty in the results obtained at each step should be assessed, along with a characterisation of the overall uncertainty in applying the safety assessment approach. Uncertainty can be expressed in qualitative, semi-quantitative or quantitative terms, depending on the nature of the data. Detailed guidance on the identification and characterisation of uncertainties has been provided by EFSA [24]. In Table 2 we have collected uncertainties identified on each stage of the ab initio workflow, as an example of how to evaluate whether we are over or underestimating the risk.

**Table 2:** Table of Uncertainties, to list information identified at each stage of the ab initio workflow for which further reasoning on over- and underestimation of risk could be considered.

Workflow Element	Information/Data
*TIER 0*	
Use Scenario(s)	12.5% content of chemical x in a body lotion applied on whole body (female, 60 kg). An average exposure assuming 100% skin penetration of a body lotion applied twice a day is estimated to 145 mg/kg/day (95th percentile of distribution for European consumers in [39], corresponding to 18.1 mg/kg/day of chemical x
Chemical Identity	Structure quality high (taken from Cosmos DB)
Existing Data	None
Exposure Assessment (exposure estimates across sectors and modelling of aggregate exposure)	Data gap
Tier 0, risk characterisation step 1: TTC applicability	Based on use scenario the exposure is too high for applying a TTC approach.
Analogues, suitability assessment and existing data	Qualitative contribution to hypothesis for target organ in a weight of evidence approach.
Tier 0, risk characterisation step 1: read-across	Analogues identified in Fig. 2. Data on safrole is available. Similarity index > 0.65. The data was not read-across to chemical X.
**TIER 0** Overall uncertainty estimation based on considerations made for individual elements	
Systemic bioavailability (target organs, internal concentration)	The PBK model show relevant doses in fatty tissue, kidney and liver. 95% confidence intervals for chemical X concentrations in the liver, blood and fat tissues of a consumer population from MC simulation.
MoA prediction based on *in silico* methods	Some models providing a qualitative indication that PPAR activation is a relevant MoA; generally, the weakness of *in silico* screens is whether the selected (available) *in silico* models are excluding any essential information on possible biological interaction.
MoA prediction based on *in vitro* screening assays	The *in vitro* data (Methods 4 and 5 in Table 1) show a strong enrichment for AOPs associated with steatosis, reproductive dysfunction and adipogenesis. The omics data show concern for hepatotoxicity including fibrosis. The dose concentrations in all *in vitro* experiments are assumed to be in a different order of magnitude compared to the actual *in vivo* exposure, for example due to binding to serum, proteins and plastics.
MoA hypothesis generation	Evaluating the PBK, *in vitro* and *in vivo* data together, liver toxicity is a relevant target organ and approximate points of departure can be set based on the available *in vitro* data from methods 4 and 5 in Table 1.
Coverage of other possible MoAs besides hepatotoxicity	Biological space too limited in the selection of cell lines/types.
**TIER 1** Overall uncertainty estimation based on considerations made for individual elements	It is assumed that the actual dose for adverse effects can be better determined.
Targeted testing (including robustness, reproducibility and relevance of new methods being used including exposure treatment)	Method 13, 14, 18 and 19 in Table 2. Indication of fibrosis and steatosis. Points of departures identified but could be further refined.
Biokinetic refinements of Points of Departure (Requires refined PBPK, *in vitro* dosimetry and mass balance measurements)	Assuming 2.1% of skin penetration, and 10% availability of the dose in experiments with cells leads to reduce over estimation in Tier 0, Tier 1 and 2.
*In vitro* to *in vivo* extrapolation	Estimate of dermal dose based on internal dose still need further development.
**TIER 2** Overall uncertainty estimation based on considerations made for individual elements	

### Final risk assessment or summary on insufficient information

When the complete data base is assessed several outcomes are possible.

• The data are considered complete and robust enough to be applied with an estimated uncertainty factor, resulting in a deterministic safety assessment assuming that certain key events lead to adverse outcome.
• The data are considered complete and robust enough to be applied with an estimated uncertainty factor, resulting in a probabilistic safety assessment estimating the possibility of adverse outcome based on the probability that certain key events might lead to a specific outcome.
• There might be too little evidence/much uncertainty to support a safety assessment, but then a gap analysis will be useful and will in itself be valuable to address to encourage further progress. In this case it could also again be evaluated whether there was any possibility to apply read-across based on strengthened evidence from alternative methods applied.
• The data are providing contradictory results to what was expected from the hypothesis. The hypothesis must be re-visited, it can also be concluded that it was actually not possible to set up a hypothesis that framed the problem enough to be able to make an adequate assessment. Also in this case it is necessary to further evaluate what additional data would have been needed, and whether there would be a method to achieve it, e.g. identify the need of additional modelling tools at Tier 1 level.

In the case of chemical x, we have summarised all possible points of departure and the estimates of concentrations in the liver from the PBK models in the Fig. 5. Fig. 5 plots the resulting points of departure for chemical x from different *in vitro* assays that were performed during the SEURAT 1 initiative. It must be appreciated that in the context and time available in the SEURAT-1 initiative the obtained information could not be completed in a way as required for e.g. regulatory consideration, however the development of the procedure and workflow were the main aim. These results show that depending on the assay the results can differ significantly of about 4 orders of magnitude, demonstrating that some assays are more sensitive to this chemical than others. The *in vitro* point of departure were plotted against the PBK model simulations, showing the potential real human concentration (physiologically relevant) of chemical x circulating in the blood stream and reaching the liver applying 100% absorption and the refined 2.1%; this information will help in refinement of *in vitro* testing and interpretation of results.

## Discussion

This general workflow was developed as a means of structuring knowledge and data in a logical sequence for an integrated safety assessment relying specifically on alternative methods and specifically taking into account exposure considerations and kinetics. The safety assessment begins with Tier 0, where the exposure scenario and chemical identity are defined and data are collected. Two exit points where identified, i) applying the TTC approach, ii) performing a read-across assessment taking into account similar substances. When TTC or read across cannot be performed, the assessment continues to Tiers 1 and 2, which define the ab initio assessment. Exposure considerations and Physiologically Based Kinetic models are important to define the target organs and internal concentrations applicable as well as to set the appropriate concentrations for the targeted testing. Data from alternative methods are collected under Tier 1 to better understand possible modes-of-action, while Tier 2 is targeted (*in vitro*) testing based on the MoA hypothesis set up under Tier 1.

The case study selected using chemical x highlights the challenge in integration of multiple data streams for safety assessment. It provides progress on how to proceed to infer a mode-of-action using a combination of *in silico*, high throughput and high content data. The use of biological and chemical sub-structure similarity screens can provide some anchoring to build confidence and give clues as to adverse outcomes on the organism level. To provide confidence in the assessment, uncertainty should be identified and evaluated for the different steps of the workflow, the methods and data contributing. Strategies for the assessment of data quality and uncertainties have been discussed in Klimisch et al. [51], Schultz et al. [80] and EFSA [24]. If the uncertainty analysis at the end highlights still too much uncertainty, there will not be a final decision on safe use, but the assessment will have identified the remaining information gaps and can make recommendations on further specific targeted *in vitro* testing or needs for relevant and reliable methods.

Skin sensitization, adipose accumulation and kidney toxicity were recognised as potential concerns during the screening in Tier 1, but we focused on liver in Tier 2, due to the focus of the methods available for testing developed within SEURAT-1. It should be recognised that other organs were not excluded and in a “real” risk assessment even broader search for target organs and mechanisms would have been considered in Tier 1.

In Tier 0 it is possible to identify exit points from the ab initio workflow and consider applying either TTC or read-across assessment. In Tier 1 it can be considered to apply an internal TTC value. This would be a more informed TTC approach built on defining internal threshold values, which would be a more relevant approach compared to the traditional TTC not including biokinetics. An internal TTC could imply to carry out PBK modelling for forward dosimetry, converting oral, dermal or inhalational exposure values into an internal (e.g. blood, or target organ) concentration. This internal concentration would then be compared with pre-defined internal threshold values to determine whether there is cause for concern, or whether the likelihood of adverse systemic effects is negligible. The internal threshold values could be derived from the traditional (external) TTC values by using computational tools (e.g. *in silico* models for bioavailability; Partosch et al. [72]) that can be used to convert the chemical specific external doses (i.e. NOAELs) in the TTC database to an estimated internal exposure. The impact of metabolism will be critical to understand in this context because it provides information regarding what is the toxicologically relevant chemical species (i.e. parent or metabolite) that should be represented in the TTC distribution.

Internal TTC values could also be derived from a sufficiently large and representative dataset of relevant *in vitro* points of departure (e.g. lowest *in vitro* concentration active in a metabolically competent system) but most likely it will be a combination of the two, with the *in vitro* points of departure being used to corroborate the model predictions.

This case study was also discussed in the context of the OECD initiative on Integrated Approaches for Testing and Assessment (IATA) Case Studies Project aimed to increase experience with the use of IATA (http://www.oecd.org/chemicalsafety/risk-assessment/iata-integrated-approaches-to-testing-and-assessment) to constitute examples of predictions that are fit for regulatory use [69], and it was recognised as a way to structuring knowledge and data in a logical sequence for an integrated chemical safety assessment relying on new approach methods starting from exposure based considerations.

## Conclusions

This general “ab initio” workflow was developed as a means of structuring knowledge and data in a logical sequence for an integrated safety assessment applying non animal methods. We believe that this workflow could be the basis for a full risk assessment and is aiming to provide a tool to guide the evaluation through the different steps to be considered and enable and gain confidence in decision making. The workflow is general enough to cover different types of chemicals, endpoints and exposure scenarios.

## Footnotes

1 PBK is used as a general term and refers to Physiologically Based Kinetic models. Synonyms found in the literature and in use in the scientific domain are PBPK (Physiologically Based PharmacoKinetic), PBTK (Physiologically Based ToxicoKinetic), PBBK (Physiologically Based BioKinetic models).
