# Supplementary material for: Ab initio chemical safety assessment: A workflow based on exposure considerations and non-animal methods
Source: Comput Toxicol. 2017 Nov;4:31–44. doi: 10.1016/j.comtox.2017.10.001 (PMC5695905; doi:10.1016/j.comtox.2017.10.001)
Supplement: Supplementary data 1 [file mmc1.docx]

Supplementary material

Ab initio chemical safety assessment:

a workflow based on exposure considerations and non-animal methods

Elisabet Berggren 🖂^1^, Andrew White^2^, Gladys Ouedraogo^3^, Alicia Paini^1^, Andrea-Nicole Richarz^1^, Frederic Y. Bois^4^, Thomas Exner^5^, Sofia Leite^6^, Leo A. van Grunsven^6^, Andrew Worth^1^ and Catherine Mahony^7^

^1^Chemical Safety and Alternative Methods Unit, & EURL ECVAM, Directorate F – Health, Consumers and Reference Materials, Joint Research Centre, European Commission, Ispra, Italy

🖂: [elisabet.berggren@ec.europa.eu](mailto:elisabet.berggren@ec.europa.eu);

^2^Unilever PLC, Bedford, United Kingdom;

^3^L’Oreal Research & Innovation, Aulnay sous bois, France;

^4^INERIS, Verneuil-en-Halatte, France;

^5^Douglas Connect, Basel, Switzerland;

^6^Liver Cell Biology Laboratory, Vrije Universiteit Brussel, Brussels, Belgium;

^7^Procter & Gamble, Egham, United Kingdom

Table SM1. Physiological Parameters used to build the human chemical x PBK model, also reported the values for Safrole as comparison.

|  | Safrole^a,b^ | chemical x^b^ |
| --- | --- | --- |
| **Tissue to blood partition coefficients:**  Liver  Lung  Kidney  Fat  Rapidly perfused  Slowly perfused  Viable Skin/stratum corneum  Viable Skin/blood  Transfer rate from stratum corneum to viable skin (ml/(h.cm^2^) | 6.7  6.7  6.7  106  6.7  4.2 | 8.77  8.77  6.69  113.7  12.10  5.40  0.1  12.1  0.1782 |
| **Physiological parameters:**  Body weight (kg)  **Fraction of body weight (L):**  Liver  Kidney  Fat  Blood  Rapidly perfused  Slowly perfuse  Cardiac output (L/h/KG bw^0.74^)  **Percentage of cardiac output:**  Liver  Kidney  Fat  Rapidly perfused  Slowly perfuse  **Skin layers thickness (cm):**  Stratum corneum  Viable skin  Surface area exposed (cm): | 60  2.6  0.8  21.4  7.9  6.6  51.7  15  22.7  17.5  5.2  29.8  24.3  0.001  0.05  15670 | |

a, Martati et al., 2012; b. DeJongh et al 1997.

*
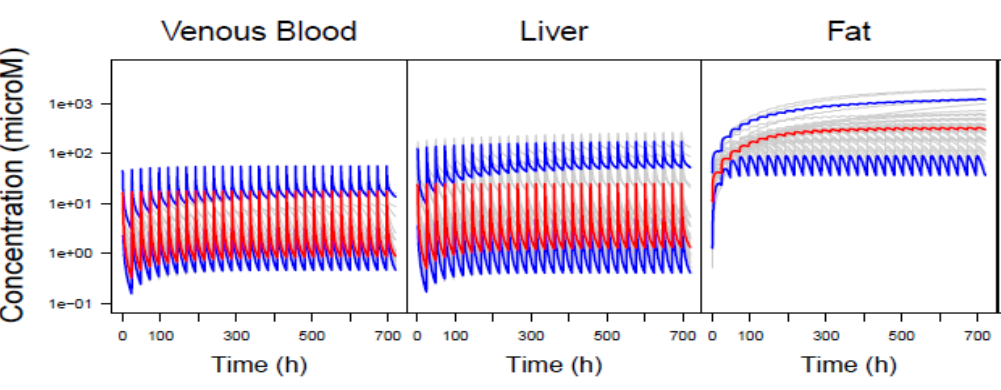
*
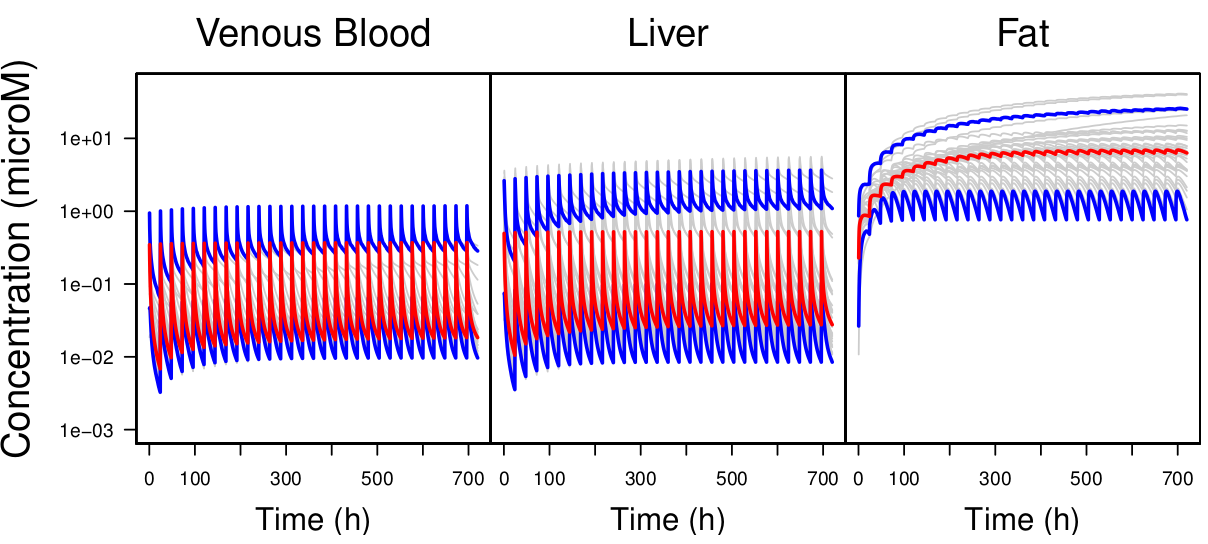


Figure SM1: PBK model simulations of the mean (red curves) and 95% confidence bounds (blue) for key-tissue concentrations of chemical x in 10000 random human subjects. A twice daily dermal exposure to a body lotion containing 12.5% chemical x (2.1% of which being available for absorption, bottom panel) was simulated for 30 days, assuming 100% skin absorption (Top panel) . The random subjects were generated by Monte Carlo simulations.

# Table SM2: Physico-Chemical properties for chemical x that are used to run the Virtual Cell Based Assay

| Chemical name | CAS | MW | Boiling point | Log P/LogKow | Henry constant | Air degradation | Water degradation | Atomic diffusion | Molar Volume |
| --- | --- | --- | --- | --- | --- | --- | --- | --- | --- |
| chemical x | 51-03-6 | 338.438 | 180°C | 4.75 Exp.l | 3.641E-006 atm-m3/mole EPIsuite | 2.4 h | 900h | 359.9 | 317.4±3.0 cm3 |
| Adjusted values to fit units for VCBA model | | | | | | | | | |
| chemical x | 51-03-6 | 338.438 | 180°C | 4.75 | 3.69E-01  Pa -m3/mole | 8.02254E-05  1/s | 2.1393E-07  1/s | 359.9 | 282.935  cm3/mole |

Table SM3 VCBA simulation for HepaRG cells treated with chemical x

| Fate model compartments | Medium | Headspace | Dissolved | Plastic | Protein | Lipid |
| --- | --- | --- | --- | --- | --- | --- |
| Chemical x% as compared to 100% in medium | 100 | 0.0005 | 3.645 | 14.1 | 31.9 | 50.4 |

Table SM4. Results of *in silico* profile predictions for chemical x.

| **Endpoint** | **Tool/Assay** | **Outcome** |
| --- | --- | --- |
| **Carcinogenicity** | CAESAR in silico model | carcinogen |
| **Mutagenicity** | CAESAR in silico model | Non mutagen |
|  | SarPy model | Non mutagen |
| **Developmental toxicity** | CAESAR in silico model | predicted non-toxic |
| **Skin sensitization** | CAESAR in silico model | sensitizer |
| **Hepatotoxicity structural alerts** | COSMOS in silico tools | 0 hits |
| **Phospholipidosis structural alerts** | COSMOS in silico tools | 0 hits |
| **Mitochondrial toxicity structural alerts** | COSMOS in silico tools | 1 hit |
| **Nuclear receptor binding** | COSMOS in silico tools | 7 hits (AHR, AR, ER, GR, PR, THR, PXR) |
|  | COSMOS virtual screening procedure | PPARγ full agonist |
|  | COSMOS in silico tools | LXR likely not a target |
| **Protein binding** | COSMOS in silico tools | 0 hit |
| **DNA binding** | COSMOS in silico tools | 0 hit |
| **Metabolic classification** | Molecular Network public set of 25 generic metabolic classes (generic set) | 7 classes activated, |
